# Phosphomannomutase deficiency (PMM2-CDG): ataxia and cerebellar assessment

**DOI:** 10.1186/s13023-015-0358-y

**Published:** 2015-10-26

**Authors:** Mercedes Serrano, Víctor de Diego, Jordi Muchart, Daniel Cuadras, Ana Felipe, Alfons Macaya, Ramón Velázquez, M. Pilar Poo, Carmen Fons, M. Mar O’Callaghan, Angels García-Cazorla, Cristina Boix, Bernabé Robles, Francisco Carratalá, Marisa Girós, Paz Briones, Laura Gort, Rafael Artuch, Celia Pérez-Cerdá, Jaak Jaeken, Belén Pérez, Belén Pérez-Dueñas

**Affiliations:** Neuropediatric Department, Hospital Sant Joan de Déu, U-703 Centre for Biomedical Research on Rare Diseases (CIBER-ER), Instituto de Salud Carlos III, Passeig Sant Joan de Déu, 2. 08950 Esplugues, Barcelona, Spain; Radiology Department, Hospital Sant Joan de Déu, U-703 Centre for Biomedical Research on Rare Diseases (CIBER-ER), Instituto de Salud Carlos III, Barcelona, Spain; Clinical Biochemistry Department, Hospital Sant Joan de Déu, U-703 Centre for Biomedical Research on Rare Diseases (CIBER-ER), Instituto de Salud Carlos III, Barcelona, Spain; Statistics Department, Fundació Sant Joan de Déu, Barcelona, Spain; Grup de Recerca en Neurologia Pediàtrica, Institut de Recerca Vall d′Hebron, Universitat Autònoma de Barcelona, Secció de Neurologia Pediàtrica, Hospital Universitari Vall d′Hebron, Barcelona, Spain; Neurology Department, Hospital Universitario La Paz, Madrid, Spain; Neurology Department, Hospital General de Sant Boi, Parc Sanitari Sant Joan de Déu, Sant Boi, Barcelona, Spain; Neurology Department, Hospital Sant Joan d’Alacant, Alicante, Spain; Hospital Clinic-IBC, IDIBAPS, Instituto de Salud Carlos III, U-737 Centre for Biomedical Research on Rare Diseases (CIBER-ER), Barcelona, Spain; Centro de Diagnóstico de Enfermedades Moleculares (CEDEM), Universidad Autónoma de Madrid (UAM), U-746 Centre for Biomedical Research on Rare Diseases (CIBER-ER) Madrid, Instituto de Salud Carlos III, IdiPAZ, Madrid, Spain; Center for Metabolic Disease, KULeuven, Leuven, Belgium

**Keywords:** Cerebellum, Congenital disorders of glycosylation, Developmental disorders, Gait disorders/ataxia, MRI, Neuropsychological assessment

## Abstract

**Background:**

Phosphomannomutase deficiency (PMM2-CDG) is the most frequent congenital disorder of glycosylation. The cerebellum is nearly always affected in PMM2-CDG patients, a cerebellar atrophy progression is observed, and cerebellar dysfunction is their main daily functional limitation. Different therapeutic agents are under development, and clinical evaluation of drug candidates will require a standardized score of cerebellar dysfunction. We aim to assess the validity of the International Cooperative Ataxia Rating Scale (ICARS) in children and adolescents with genetically confirmed PMM2-CDG deficiency. We compare ICARS results with the Nijmegen Pediatric CDG Rating Scale (NPCRS), neuroimaging, intelligence quotient (IQ) and molecular data.

**Methods:**

Our observational study included 13 PMM2-CDG patients and 21 control subjects. Ethical permissions and informed consents were obtained. Three independent child neurologists rated PMM2-CDG patients and control subjects using the ICARS. A single clinician administered the NPCRS. All patients underwent brain MRI, and the relative diameter of the midsagittal vermis was measured. Psychometric evaluations were available in six patients. The Mann–Whitney *U* test was used to compare ICARS between patients and controls. To evaluate inter-observer agreement in patients’ ICARS ratings, intraclass correlation coefficients (ICC) were calculated. ICARS internal consistency was evaluated using Cronbach’s alpha. Spearman’s rank correlation coefficient test was used to correlate ICARS with NPCRS, midsagittal vermis relative diameter and IQ.

**Results:**

ICARS and ICARS subscores differed between patients and controls (*p* < 0.001). Interobserver agreement of ICARS was “almost perfect” (ICC = 0.99), with a “good” internal reliability (Cronbach’s alpha = 0.72). ICARS was significantly correlated with the total NPCRS score (r_s_ 0.90, *p* < 0.001). However, there was no agreement regarding categories of severity. Regarding neuroimaging, inverse correlations between ICARS and midsagittal vermis relative diameter (r_s_ −0.85, *p* = 0.003) and IQ (r_s_ −0.94, *p* = 0.005) were found. Patients bearing p.E93A, p.C241S or p.R162W mutations presented a milder phenotype.

**Conclusions:**

ICARS is a reliable instrument for assessment of PMM2-CDG patients, without significant inter-rater variability. Despite our limited sample size, the results show a good correlation between functional cerebellar assessment, IQ and neuroimagingFor the first a correlation between ICARS, neuroimaging and IQ in PMM2-CDG patients has been demonstrated.

## Background

Congenital disorders of glycosylation (CDG) comprise some 100 genetic disorders caused by impaired synthesis of glycoconjugates [1, 2]. Mammals have eight major glycosylation pathways in the endoplasmic reticulum and Golgi, and the N-linked glycosylation pathway is by far the best studied [3, 4].

Phosphomannomutase deficiency (PMM2-CDG, previously CDG-Ia), is the most frequent congenital disorder of N-linked glycosylation and accounts for approximately 80 % of all diagnosed patients since it was described in 1980 [5]. PMM2-CDG is caused by mutations in *PMM2* (*#601785OMIM), encoding an enzyme that catalyzes the second step in the N-glycosylation pathway, the conversion of mannose-6-phosphate to mannose-1-phosphate.

The CNS and peripheral nervous system are prime targets of PMM2-CDG deficiency, but many other systems are affected, as in other CDG [2].

Early clinical signs of PMM2-CDG are usually abnormal fat distribution, inverted nipples, strabismus and hypotonia. Infants develop ataxia, psychomotor delay and extraneurological manifestations, including failure to thrive, enteropathy, hepatic dysfunction, coagulation abnormalities and cardiac and renal involvement. The phenotype is extremely variable: some patients develop a severe systemic illness leading to early death [6, 7], and some patients show milder forms with variable systemic and mild neurological involvement [2, 8].

Cerebellar involvement is a common feature of PMM2-CDG. At birth, most children have marked hypotrophy of the vermis and cerebellar hemispheres, followed by progression towards atrophy [9, 10]. However, in mild cases, brain MRI may be normal [8]. Many key neurological symptoms found in PMM2-CDG patients can be explained by this cerebellar syndrome (ataxia, dysmetria, tremor, abnormal eye movements, dysarthria, and cognitive deficits or low intelligence quotient (IQ)), leading to major disability.

The recently validated Nijmegen Pediatric CDG Rating Scale (NPCRS) based on the Newcastle Paediatric Mitochondrial Disease Scale, evaluates multisystemic data, including neurological involvement [11]. However, this scale does not specifically assess cerebellar symptoms.

The International Cooperative Ataxia Rating Scale (ICARS) is a neurologist-completed scale developed to assess cerebellar ataxia [12], that has been recently validated in healthy children above 4 years of age [13]. ICARS has been used in some genetic diseases such as Friedreich ataxia, coenzyme Q deficiency and others [14, 15]. However, no standardized tools have been used to evaluate cerebellar dysfunction in PMM2-CDG patients.

In the present study, we aim to assess the validity of the ICARS in children and adolescents with genetically confirmed PMM2-CDG deficiency. ICARS results are compared with NPCRS, neuroimaging, IQ and molecular data.

## Methods

Eligible patients included children and adolescents older than four years of age with a molecular diagnosis of PMM2-CDG. Exclusion criteria were severe cognitive impairment or behavioral problems precluding complete administration of the scales.

A pediatric age-matched control population was selected. The control sample included children older than four years recruited by open advertisement or from children attending the Hospital Sant Joan de Déu (HSJD) outpatient clinic for mild, untreated tensional headache. Exclusion criteria were cognitive or behavioral problems.

ICARS involves a 100-point rating scale with higher scores denoting more evident clinical abnormalities (https://commondataelements.ninds.nih.gov/Doc/NOC/International_Cooperative_Ataxia_Rating_Scale_NOC_Link.pdf) [12]. ICARS includes subscores for posture and gait (0–34), kinetic functions (0–52), speech abnormalities (0–8) and oculomotor function (0–6). Three independent child neurologists rated PMM2-CDG patients simultaneously with the ICARS. All of the examinations were videotaped following a standardized protocol for educational and revision purposes. The NPCRS was administered by one of the three child neurologists on the same day as the ICARS assessment.

**Fig. 1:**
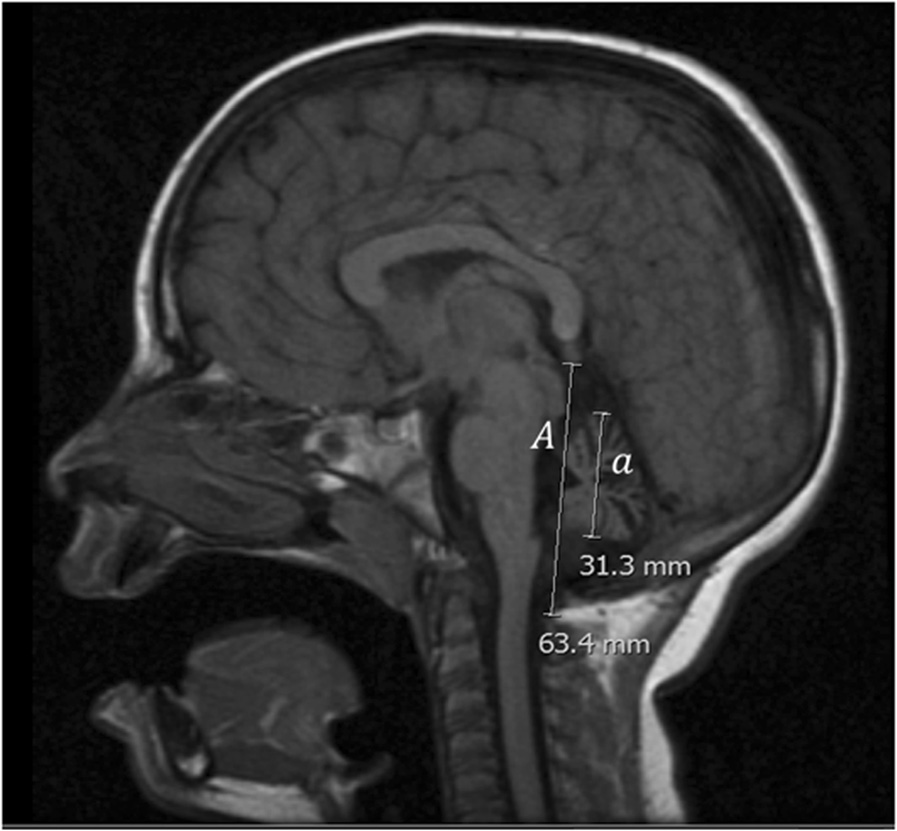
Midsagittal vermis relative diameter (MVRD). Legend: Midsagittal vermis relative diameter (MVRD) was calculated using a midsagittal section and measuring total posterior cranial fossa diameter (a linear segment from the posterior commissura to the occipital bone at the foramen magnum) and the largest axial diameter of the cerebellum parallel to the previous linear segment. The fraction (cerebellum diameter/total posterior cranial fossa diameter) is used to express the proportion of both values: $MVRD=100\frac{a}{A}$. The image represents MVRD calculation of patient 5, with a MVRD result of 49 %

Brain MRI exams included T1 and T2-weighted, diffusion-weighted and FLAIR sequences. An adapted measurement procedure similar to the classic fetal cerebellum measurements was applied [16, 17]. The relative diameter of the midsagittal vermis was calculated using a midsagittal section and measuring total posterior cranial fossa diameter in a linear segment from the posterior commissura to the opisthium and the largest sagittal diameter of the cerebellum parallel to the previous linear segment. The ratio of the cerebellum diameter over the total posterior cranial fossa diameter was used (Fig. 1). Only those patients with MRI studies performed within the two years preceding ICARS assessment were included in this analysis (*n* = 9).

Biochemical and molecular studies were carried out in all PMM2-CDG patients enrolled. Genetic analysis was performed in the CEDEM-UAM in Madrid. Total mRNA and genomic DNA were isolated from venous whole blood or patient-derived fibroblasts using a MagnaPure system following the manufacturer’s protocol (Roche Applied Science, Indianapolis). Mutational analysis was performed by genomic DNA analysis both in patients’ and parents’ samples to assure that both changes are on different alleles and to rule out the presence of a large genomic rearrangement. In some cases the effect on splicing was analyzed by cDNA profile analysis. The primers used for cDNA and genomic DNA amplifications were designed using the ENSEMBL database (http://www.ensembl.org/index.html, ENSG00000140650) and GenBank accession number NM_000303.2. Segregation genetic studies were performed in all patients. Maternal isodisomy was detected in patients 12 and 13 by SNP array analysis (Infinium CytoSNP-850 K BeadChip).

For intelligence evaluation, six patients received structured neuropsychological testing; their global IQ, obtained from WISC-IV-R or K-BIT tests, was used for analysis.

Ethical permission for the study was obtained from the Research & Ethics Committee of the HSJD. Parents gave their written informed consent and children/adolescents gave their assent. Samples were obtained in accordance with the Helsinki Declaration of 1964, as revised in October 2013 in Fortaleza, Brazil.

Statistical analysis was performed using SPSS V.22.0 (Armonk, NY: IBM Corp.). The Mann–Whitney *U* test was used to compare ICARS and ICARS subscales between patients and controls. To evaluate inter-observer agreement in patients’ ICARS and ICARS sub-scales, intraclass correlation coefficients (ICC) were calculated. Internal consistency of the ICARS scale and its sub-scales was evaluated using Cronbach’s alpha. Spearman’s rank correlation coefficient test was used to study the relationship between the ICARS and the NPCRS, mid-sagittal vermis relative diameter and IQ. To avoid the confounding effect of age in the patient sample, a new ICARS value for every patient was obtained subtracting the mean values of the ICARS found in our control population at different ages. All statistical tests were two-sided. *P*-values <0.05 were considered significant.

## Results

Thirteen PMM2-CDG children (mean age 9.3 years (SD 3.5; range [4.9-16.2]); five males, eight females) were included in the study. One teenager affected by PMM2-CDG was excluded due to severe cognitive impairment that precluded the assessment of ICARS. Tables 1 and 2 show clinical, radiological and molecular data, ICARS and NPCRS results. Twenty-one children between the ages of four and fifteen years were included in the study as control subjects (mean age 8.41 years (SD 3.2; Range [4.0-14.3]); 13 males, eight females). Eleven were recruited by open advertisement, and ten were outpatients.

**Table 1:** Molecular findings and multiorgan characteristics

Patient/Sex	1 Male	2 Female	3 Female	4 Male	5 Male	6 Female	7 Female	8 Male	9 Female	10 Female	11 Female	12 Female	13 Male
Molecular findings	p.E93A/p.R141H	p.R123Q/p.C241S	p.F157S/p.C241S	p.L32R/p.R141H	p.R162W/c.523 + 3A > G	p.F157S/p.R162W	p.P113L/p.D209G	p.C241S/p.R141H	p.T237M/c.640-9 T > G	p.P113L/c.353C > T + c.550C > A	p.R123X/p.I153T	p.P113L/p.P113L	p.E139K/ p.E139K
Age at evaluation/age at onset	15 years/21 months	6 years/9 months	8 years/8 months	7 years/Newborn	10 years/3 months	10 years/8 months	11 years/Newborn	5 years/2.5 years	11 years/8 months	8 years/7 months	16 years/Newborn	6 years/3 months	11 years/ Newborn
Dysmorphic features	Large normal setting ears	Almond slanted eyes	No	Anteverted ears	Inverted nipples	Lipodystrophy	Almond slanted eyes, lipodystrophy	No	Almond slanted eyes	Almond slanted eyes, low anteverted ears, lipodystrophy	Almond slanted eyes, thick lips, pronounced cheekbones, lipodystrophy	Almond slanted eyes, broad face, large mouth, lipodystrophy,	Almond slanted eyes, broad face, large mouth, lipodystrophy
Cardiac involvement	No	No	No	No	No	No	No	No	No	No	No	PDA	Truncus arteriosus
Digestive involvement	Diarrhea, hypoglycemia	No	No	Vomits	No	↑ALT	↑ALT, AST (only perinatal period)	No	↑ALT, AST	Growth failure, hypoglycemia	Vomits, growth failure, diarrhea	↑ALT, AST	Failure to drive
Coagulation	↓ AT-III, PC	↓ AT-III, PC, PS	↓ AT-III, PC	↓ AT-III, PC	Normal	↑ PT; ↓AT-III, PS	↓ PC, PS	No	↓FXI	↑PT, aPTT,	↓ aPTT	↓ AT-III	↑PT
										↓AT-III, PC, PS			
Endocrine involvement	No	No	No	No	No	No	Hypergonadotropic hypogonadism	No	No	Mild ↑TSH, hypoglycemia	Delayed puberty	ND	No
Kidney involvement	Nephro calcinosis	No	No	No	No	No	No	No	No	↑Echogenicity, nephrolithiasis	Proteinuria	No	No
Eye abnormalities	Strabismus, pigmentary retinopathy	Strabismus upgaze deviation	Strabismus	Strabismus, hypermetropia, astigmatism	Strabismus, saccadic movements hypermetropia	Strabismus, hypermetropia	Strabismus, hypermetropia, astigmatism, abnormal ERG	No	Strabismus	Strabismus, upgaze deviation, saccadic movements	Strabismus, upgaze deviation, retinopathy, hypermetropia	Strabismus, hypermetropia	Strabismus

*PDA* Persistent ductus arteriosus, *ALT* Alanine transaminase, *AST* Aspartate transaminase, ↑ Increased values, ↓ Decreased values, *AT-III* Antithrombin III, *PC* Protein C, *PS* Protein S, *FXI* Coagulation factor XI, *aPTT* Activated partial thromboplastin time, *FIX* Coagulation factor IX, *PT* Prothrombin time, *ND* No data available, *TSH* Thyroid stimulating hormone

**Table 2:** Molecular findings and neurological evaluation (neuroimaging, IQ, NPCRS and ICARS results)

Patient/Sex	1 Male	2 Female	3 Female	4 Male	5 Male	6 Female	7 Female	8 Male	9 Female	10 Female	11 Female	12 Female	13 Male
Molecular findings	p.E93A/p.R141H	p.R123Q/p.C241S	p.F157S/p.C241S	p.L32R/p.R141H	p.R162W/c.523 + 3A > G	p.F157S/p.R162W	p.P113L/p.D209G	p.C241S/p.R141H	p.T237M/c.640-9 T > G	p.P113L/c.353C > T + c.550C > A	p.R123X/p.I153T	p.P113L/p.P113L	p.E139K/p.E139K
Age at evaluation	15 years	6 years	8 years	7 years	10 years	10 years	11 years	5 years	11 years	8 years	16 years	6 years	11 years
Peripheral neuropathy	Motor-sensory axonal neuropathy	No NC/↓DTR	No NC/↓DTR	No	No NC/↓DTR	Slow nerve conduction	No	No NC/↓DTR	No NC/↓DTR	Slow nerve conduction velocity	Slow nerve conduction velocity	No NC/↓DTR	No NC/↓DTR
Epilepsy	No	No	No	No	No	No	No	No	No	No	No	No	Yes
Psychometric evaluation	IQ 91 (WISC-IV)	ND	IQ 89 (WISC-IV)	ND	IQ 78 (K-BIT)	IQ 59 (WISC-IV)	ND	ND	ND	IQ 66 (K-BIT)	IQ 40 (K-BIT)	ND	ND
MRI findings	Mild prominence cerebellar folia	Cerebellar vermis atrophy	Global cerebellar atrophy	ND	Global cerebellar atrophy	Global cerebellar atrophy	Global cerebellar atrophy	Global cerebellar atrophy	Cerebellar vermis atrophy	Global cerebellar atrophy, juxtacortical signal change	Global cerebellar atrophy with pons atrophy	Global cerebellar atrophy	Global cerebellar and pons atrophy, ventricular enlargement
MRI MVRD	67 %	46 %	No recent MRI^a^	No recent MRI^a^	49 %	46 %	35 %	No recent MRI^a^	No recent MRI^a^	35 %	29 %	36 %	28 %
NPCRS	6	8	8	12	8	11	20	13	16	18	22	16	27
ICARS	4	10	17	18	23	34	47	50	60	61	62	70	79

*NC* Nerve conduction studies, *DTR* Deep tendon reflexes, *IQ* Intelligence quotient, *WISC-IV* Wechsler Intelligence Scale for Children, Fourth edition, *K-BIT* Kaufman Brief Intelligence Test, Second edition, *MVRD* midsagittal vermis relative diameter, *NPCRS* Nijmegen Pediatric CDG Rating Score, *ICARS* International Cerebellar Assessment Rating Scale

^a^See criteria in the text to include MRI in the analysis

**Fig. 2:**
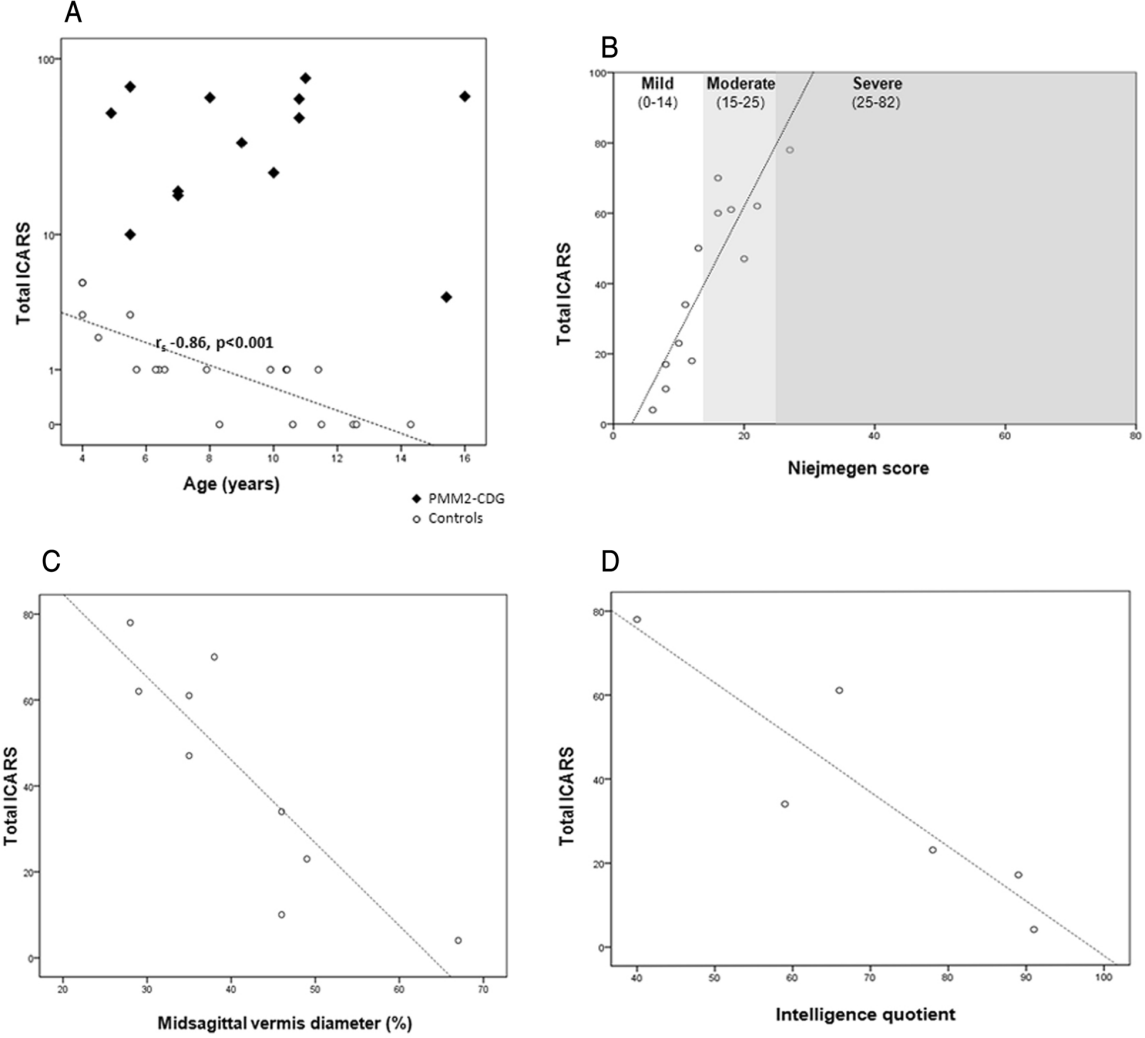
ICARS results in patients versus control subjects, and correlations with NPCRS, neuroimaging and IQ findings. Legends: **a** ICARS: Patients’ mean 41.1 vs Controls’ mean 1.3 (*p* < 0.01); Postural-gait subscore: Patients’ mean 15.3 vs Controls’ mean 0.4 (*p* < 0.01); Kinetic subscore: Patients’ mean 20.2 vs Controls’ mean 0.8 (*p* < 0.01); Dysarthria subscore: Patients’ mean 3.5 vs Controls’ mean 0.1 (*p* < 0.01); Oculomotor subscore: Patients’ mean 1.7 vs Controls’ mean 0.1 (*p* < 0.01). **b** ICARS was statistically correlated with NPCRS with a positive coefficient of correlation (r_s_ 0,90, *p* < 0.001). Regarding NPCRS sections, ICARS was correlated with Section I (Current Function) (r_s_ 0.92, *p* = 0.001), and Section III (Current Clinical Assessment) (r_s_ 0.88, *p* < 0.001) but not with Section II (System Specific Involvement) (r_s_ 0.35, *p* = 0.27). **c** ICARS is negatively correlated with midsagittal vermis relative diameter (rs −0.87, *p* = 0.003). **d** There is a negative correlation between ICARS and intelligence quotient (rs −0.94, *p* = 0.005)

ICARS and ICARS subscores were significantly different between patients and controls (patients’ mean 41.1 vs. controls’ mean 1.3; *p* = 0.01) (Fig. 2a). There was a statistically significant inverse correlation between the controls’ age and ICARS that was not present in patients (r_s_ −0.86, *p* < 0.001).

The ICCs for interobserver agreement of ICARS (three different observers) was scored “almost perfect” (ICC = 0.99), with an internal reliability when the four subscales are included scoring as “good” (Cronbach’s alpha = 0.72). Only exclusion of the oculomotor subscore would have increased Cronbach’s alpha. Inter-item evaluation in every subscale reliability was scored “excellent” for postural/gait, kinetic and dysarthria subscores (0.96, 0.94 and 0.88, respectively) and “poor” for the oculomotor subscale (0.20).

The ICARS score was positively correlated with the total NPCRS score (r_s_ 0.90, *p* < 0.001) (Fig. 2b). However, patients with high ICARS values (60, 61 and 70, respectively) were in the low-moderate category of severity according to the NPCRS, and only the most severe patient (79 total ICARS) was in the low range of the severe category in the NPCRS (Fig. 2b). Regarding the NPCRS sections, ICARS correlated with Section I (Current Function) (r_s_ 0.92, *p* < 0.001), and Section III (Current Clinical Assessment) (r_s_ 0.88, *p* < 0.001) but not with section II (System Specific Involvement) (r_s_ 0.35, *p* = 0.27).

**Fig. 3:**
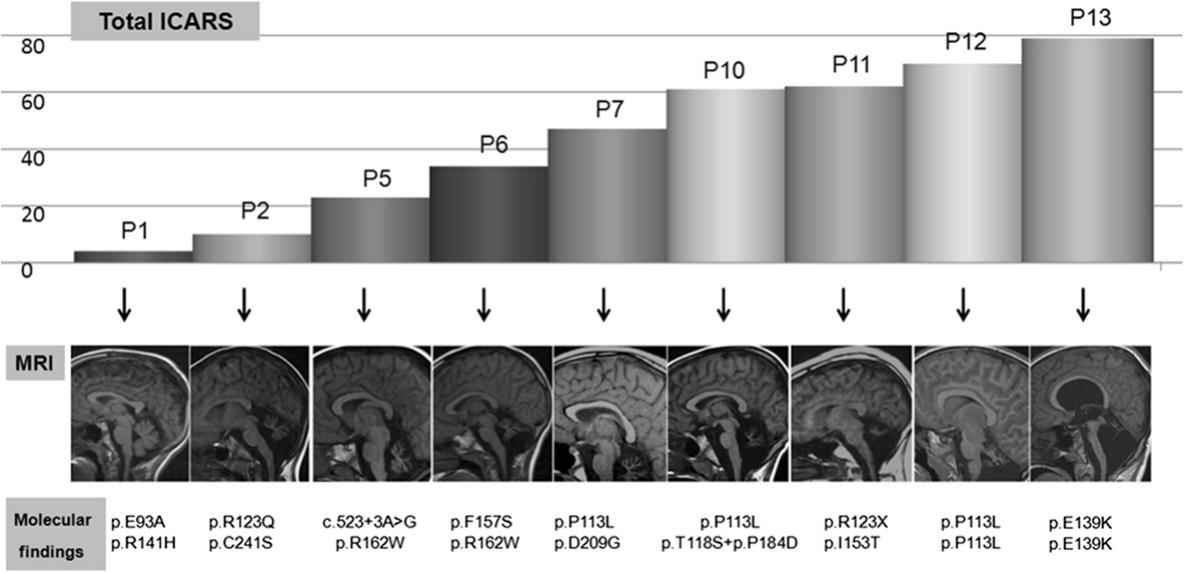
ICARS and neuroimaging findings

Regarding the genetic basis, 14 different previously described mutations were identified, and one novel mutation (c.523 + 3A > G). Eleven patients were compound heterozygous and two were homozygous for the p.P113L or p.E139K. Both patients presented a maternal isodisomy. Transcriptional profile analysis of patient 6 patient-derived fibroblasts revealed an aberrant transcript due to skipping from exon 3 to 6 (r.179_523del345, p.Val60_Ile174del). Patients bearing p.E93A, p.C241S or p.R162W mutations presented a milder phenotype with less severe ICARS and milder cerebellar findings on MRI. Patients bearing p.P113L and E139K presented the most severe ICARS and cerebellar atrophy (Fig. 3).

All patients showed different degrees of cerebellar atrophy on MRI, mostly vermian atrophy. The cerebellar atrophy was more evident in those with higher ICARS (Fig. 3). An inverse correlation between midsagittal vermis relative diameter and ICARS was found (r_s_ −0.85, *p* = 0.003) (Fig. 2c). Midsagittal vermis relative diameter was also inversely correlated with NPCRS (r_s_ −0.95, *p* < 0.001).

Concerning IQ, despite the small number of patients available, there was a negative correlation between ICARS and IQ (r_s_ −0.94, *p* = 0.005) (Fig. 2d), NPCRS and IQ (r_s_ −0.94, *p* = 0.005) and midsagittal vermis relative diameter and IQ (r_s_ −0.90, *p* = 0.037).

To avoid the confounding effect of age in the patients’ sample, a new ICARS value for every patient was obtained subtracting the mean values of ICARS found in our control population at different ages. All of the above described significant correlations between ICARS and NPCRS, MRI findings or IQ remained unchanged when the new ICARS value was introduced into the analyses.

## Discussion

ICARS is a commonly used evaluation for patients with ataxia. It is an exhaustive scale from which some other derivatives, such as BARS (Brief Ataxia Rating Scale), have been created to increase the speed of the evaluation. The value of ICARS, BARS and SARA (Scale for the Assessment and Rating of Ataxia) have recently been validated in the pediatric population above 4 years of age [13]. SARA includes a restricted number of items; for instance, oculomotor abnormalities are not included, which is a major drawback because they are frequently found in PMM2-CDG patients. The previous evidence of ICARS reliability when applied to children underlines the necessity of age correction in the interpretation of absolute ICARS results. This is particularly true in the sub-scores of posture and gait and limb coordination, as both areas are the most influenced by variation in fine and gross motor skills and change during normal neurodevelopment [13].

Regarding pediatric patients under 4 years of age, at the present time, big efforts are being performed by patients’ associations and clinicians to develop an ataxia rating score.

In the present study, differences in ICARS scores between PMM2-CDG patients and controls were important and statistically significant. There was no age effect in patient scores when a “corrected-by-age” ICARS was included in the analysis. This fact is probably due to the limited impact of age compared to the effect of the clinical phenotype.

When applying ICARS, inter-observer variability varied between “almost perfect” and “substantial”. Concerning inter-item reliability for every subscale, the worst results were obtained regarding oculomotor abnormalities. This may be explained by the fact that only three items were evaluated and by the influence of the collaboration of the patient, hampering proper scoring and data interpretation. This methodological issue should prompt reconsideration of SARA, a scale that does not rely upon evaluation of oculomotor abnormalities. However, this is a major drawback because signs of oculomotor dysfunction were frequently observed in our series of PMM2-CDG patients, and the oculomotor subscore was significantly higher in patients compared to controls (Fig. 2a).

Non-cerebellar aspects of the neurological spectrum of PMM2-CDG patients can act as potential confounders to ICARS assessment, such as muscle weakness, peripheral neuropathy, extracerebellar muscle hypotonia and cognitive impairment, increasing the final score. Sival et al. evaluated sensory ataxia and muscle weakness in children affected by Friedreich ataxia scored using ICARS [14]. They conclude that only maximal sensory ataxia and pronounced muscle weakness increase the ICARS score, reaching a maximal plateau. None of our included PMM2-CDG patients had pronounced muscle weakness or severe peripheral neuropathy. Moreover, unlike Friedreich ataxia, spinal cord abnormalities are not included in the PMM2-CDG phenotype.

Concerning cognitive impairment as a further limitation to ICARS, we facilitated the comprehension of the different maneuvers by training the patient before the final evaluation. In addition, we allowed the patients to imitate movements, as they completed the test together with their siblings or parents. The evaluators found that the patients’ comprehension was enough to understand and perform the maneuvers in thirteen PMM2-CDG patients. However, one teenager was excluded from the study due to severe cognitive impairment that precluded the assessment of ICARS. We found limb coordination and oculomotor sub-scores the more challenging items both for patients and controls.

In summary, two main limitations should be considered in a particular patient when rating a PMM2-CDG patient with ICARS; the level of cooperation/comprehension as well as sensory abnormalities and/or peripheral neuropathy. Considering that PMM2-CDG patients usually associate cognitive impairment, their neurodevelopmental quotient is relevant for the interpretation of ICARS. Finally, future longitudinal studies will be necessary to demonstrate the validity of this scale when assessing the progression of cerebellar manifestations in PMM2-CDG.

The NPCRS is a scale based on the Newcastle Pediatric Mitochondrial Disease Scale recently validated for CDG patients, reflecting cross-usefulness in these two groups of diseases with multiple organ system involvement [11]. Although NPCRS is a good tool for the global evaluation of the patient, it has poor specificity for cerebellar symptoms. In PMM2-CDG, ataxia, and, to a lesser extent, other neurological abnormalities, appear to be the main cause of the patients’ daily limitations. Therefore, the ICARS seems a more precise option when addressing functional disability in PMM2-CDG. In our study there was a good correlation between ICARS and total NPCRS results. However, the NPCRS did not seem to discriminate between moderate and severe cerebellar phenotypes.

Due to the broad mutation spectrum in the Spanish population [8], and our limited sample size, our ability to reach conclusions regarding genotype-phenotype correlations is limited. However, some conclusions can be drawn by taking into account the reported functional effects of some mutations [18], and the patient’s phenotype described in this work. Among our patients, p.E93A, p.C241S and p.R162W mutations that retain residual activity, combined with null missense mutations with no residual activity (p.R141H, p.R123Q or p.F157S) or splicing and nonsense mutations (c.523 + 3A > G and p.R123X) were observed with milder phenotypes. Therefore, these mutations p.E93A, p.C241S and p.R162W can be considered milder mutations. It is noteworthy that the two homozygous patients present the most severe phenotype. Regarding the mutation showed by patient 13, the p.E139K mutant protein retains 25 % of its residual activity, and the protein can be expressed at a sufficient level in vivo to confer residual activity compatible with life [19], being found in milder phenotypes. Concerning the mutation showed by patient 13, p. P113L mutation affects PMM2 dimerization and is present in this work in three patients associated with different phenotypes being associated with a less severe phenotype in heterozygosity [18]. Importantly, patients 12 and 13 presented a segmental maternal chromosome isodisomy [20] a genetic condition reported in chromosome 16 [21]; other sequence variants present in this homozygous region could affect the clinical features in these two patients.

An important neurological feature in PMM2-CDG is cerebellar ataxia. No clear correlation between the severity of ataxia and cerebellar imaging has been reported previously. In our sample, an apparent relationship was observed between ICARS and the severity of cerebellar atrophy because higher ICARS scores were associated with wider inter-folia spaces and lower cerebellar volumes by visual inspection. A more objective, simple and very easy measure is the midsagittal vermis relative diameter as performed on MRI studies. A negative correlation between cerebellar measures and ICARS supports a clinical and anatomical correlation; however, this simple measure can be biased by many factors. 3D segmentation and measurement of the cerebellum has proven to be most accurate in assessing cerebellar volume as has been reported in other genetic diseases [22]. Unfortunately, in the vast majority of our recruited patients, only non-volumetric MRI scans were available. Therefore, we have been forced to use a linear measurement approach similar to the classic fetal cerebellum measurements [16, 17], in order not to repeat MRI examinations for ethical reasons (most of the patients would probably need sedation). Although this method is not as precise, it is easily reproducible and is able to show gross cerebellum size anomalies in relationship to the posterior fossa size. Furthermore, in patients with more than one MRI, a progression of the cerebellum atrophy was found (data not reported). Whether this neuroimaging progression correlates with a measurable clinical worsening and whether this progression is pointing toward a therapeutic window deserves further studies.

Regarding supratentorial abnormalities, patient 13 showed lateral ventricular enlargement. This patient underwent cardiac surgery during the newborn period and suffered a prolonged cardiorespiratory arrest during the procedure. This hypoxic-ischemic event may explain neuroimaging findings aside from the expected cerebellar atrophy.

The cerebellum, while once considered a brain region principally involved in motor control and coordination, is increasingly associated with a range of neuropsychological and neuropsychiatric presentations. In patients who suffer from different cerebellar disorders, cerebellar degeneration and focal cerebellar lesions, impairments in attention, memory, executive functions and intelligence quotient demonstrate that the cerebellum likely plays a significant role in numerous higher cognitive functions such as language, cognitive and emotional functions [23]. The analysis of all of these traits in our patients was not within the scope of our present work. However, interestingly, a negative correlation between ICARS and QI was found. Again, this is a complex issue that deserves further study.

## Conclusions

In conclusion, our study demonstrates the reliability of ICARS for the assessment of cerebellar involvement in PMM2-CDG patients, showing no significant inter-rater variability. Our results suggest a correlation between cerebellar symptoms and neuroimaging findings that needs to be further explored.
